# A Study on Weight Loss Cause as per the Side Effect of Liraglutide

**DOI:** 10.1155/2022/5201684

**Published:** 2022-12-02

**Authors:** Jin Yu, Jeongmin Lee, Seung-Hwan Lee, Jae-Hyung Cho, Hun-Sung Kim

**Affiliations:** ^1^Division of Endocrinology and Metabolism, Department of Internal Medicine, Seoul St. Mary's Hospital, College of Medicine, The Catholic University of Korea, Seoul 06591, Republic of Korea; ^2^Division of Endocrinology and Metabolism, Department of Internal Medicine, Eunpyeong St. Mary's Hospital, College of Medicine, The Catholic University of Korea, Seoul 03312, Republic of Korea; ^3^Department of Medical Informatics, College of Medicine, The Catholic University of Korea, Seoul 06591, Republic of Korea

## Abstract

**Purpose:**

Liraglutide is known to have much lower weight loss effects in real clinical fields than in randomized clinical trials because of its side effects (SE) and discomfort associated with injections. This study is aimed at determining whether the side effects of liraglutide affect weight reduction and its maintenance in real-world practice.

**Methods:**

Endocrinologists conducted a retrospective chart review of data from two tertiary university hospitals. All patients who had been prescribed liraglutide at least once between January 2014 and December 2019 were included. For an average of 3 and 6 months, weight changes due to the presence or absence of SE and discontinuation (MAIN or STOP) of liraglutide were checked.

**Results:**

Only 40.8% (64/157) of the patients remained on liraglutide for 6 months; 14.7% (23/157) maintained the drug despite SEs (MAIN_SE(+)), and 40.1% (63/157) discontinued the drug despite not having SEs (STOP_SE(-)). At 3 months, there was −5.9 ± 0.6%, −7.9 ± 0.9%, −4.5 ± 0.5%, and −3.4 ± 0.6% weight reduction in the MAIN_SE(-), MAIN_SE(+), STOP_SE(-), and STOP_SE(+) groups, respectively (all *p* < 0.001 compared to the baseline). However, there were no significant differences in the weight loss between the MAIN (*p* = 0.062) and STOP (*p* = 0.204) groups. At 6 months, the weight reduction was −2.0 ± 0.5% (*p* < 0.001) in MAIN_SE(-), −2.2 ± 0.7% (*p* < 0.005) in MAIN_SE(+), −1.7 ± 0.7% (*p* < 0.01) in STOP_SE(-), and −2.0 ± 0.6% (*p* = 0.01) in STOP_SE(+), compared to baseline. SEs also caused no significant differences in weight loss between the MAIN (*p* = 0.787) and STOP (*p* = 0.694) groups.

**Conclusions:**

Our study confirmed that the side effects of liraglutide did not affect weight reduction. Moreover, in the real world, the continuous rate of liraglutide use is not high, and the weight gradually increases after 3 months. Therefore, in addition to the side effects of liraglutide, the medical staff should consider various factors that affect drug adherence, consider ways to increase compliance, and continue to ensure management so that patients can maintain their weight.

## 1. Introduction

It is important to manage overweight and obesity as they are risk factors for type 2 diabetes [1, 2]. Currently, lifestyle changes, including diet and exercise, are recommended for obesity management, along with drug-based weight loss [3]. Glucagon-like peptide-1 receptor agonists (GLP-1RA) increase insulin release, decrease glucagon secretion, and exert good blood sugar control effects. Therefore, liraglutide, the first GLP-1RA, was used as an antidiabetic drug. GLP-1RA affects satiety and suppresses appetite by delaying gastric emptying, thereby reducing weight; it is one of the drugs used to treat obesity. According to the results of several studies, the weight reduction effect of liraglutide is good, as at least a 5% weight reduction can be achieved when using liraglutide for 1 year [4–6]. However, it is associated with several side effects due to delayed gastric emptying, such as nausea and vomiting.

Reports on the weight change due to the side effects of liraglutide have been controversial. Some studies have reported that the weight reduction effect is greater than the side effects; however, some have also reported no relationship between the side effects and weight reduction effect of liraglutide [5, 7]. In addition, there is a report that no biased effect exists due to side effects as per the studies examining drug effects [8]; therefore, there is still a lot of debate in this regard [5, 9]. Since there are limitations in accurately measuring the side effects as well as their duration, few studies have been conducted on the relationship between side effects and weight reduction. The results of these studies are controversial; therefore, the effect of weight loss due to side effects is still uncertain. In addition, little is known about side effects and drug discontinuation in the real clinical field that reflect patient compliance.

Liraglutide is known to have a greater weight reduction effect than other obesity drugs [5]. However, one study reported weight recovery after the maximum effect of the drug [6]. In the real world, some patients tolerate and maintain liraglutide even if there are side effects, while there are cases where the drug is stopped for various reasons such as compliance, even if there are no side effects. There are no known studies on the compliance of liraglutide, such as side effects and drug discontinuation, in actual clinical practice. It is also unknown whether liraglutide weight loss is as effective in the real world as in previous well-known randomized control studies [10]. The purpose of this study was to investigate the degree of maintenance of weight loss and the effects of side effects on weight loss after liraglutide prescription in actual clinics.

## 2. Materials and Methods

In this study, among the patients who visited Seoul St. Mary's Hospital and Eunpyeong St. Mary's Hospital, from January 2014 to December 2019, those who had been prescribed liraglutide at least once were followed up for an average of 6 months.

### 2.1. Study Population and Design

The index date was designated as the date on which liraglutide was prescribed. Personal information such as age, sex, weight, and body mass index (BMI) of the patients within 2 weeks before the index date was collected. Baseline laboratory results of blood glucose, HbA1c, blood urea nitrogen, and creatinine levels, along with liver function tests (aspartate aminotransferase, alanine aminotransferase, alkaline phosphatase, and gamma-glutamyl transpeptidase), and lipid profiles (total cholesterol, triglycerides, HDL cholesterol, and LDL cholesterol) were collected. In addition, the history of hypertension, prediabetes, diabetes, dyslipidemia, fatty liver, thyroid disease, and allergies was investigated. All side effects that the patients complained about were confirmed, including gastrointestinal (GI) problems caused by the mechanism of action of liraglutide. Additionally, history of psychiatric disorders or psychiatric treatment was assessed.

A visit 2–4 months (average 3 months) from the index date was designated as visit 1, and a visit 5–7 months (average 6 months) later was designated as visit 2. The patient's weight was recorded at each visit, and the change from the baseline weight was calculated. If there were multiple visits within 2–4 months, the lowest weight was selected. If there were multiple visits within 5–7 months of the last visit, the weight closest to 6 months was selected. Additionally, the maximum dosage of liraglutide was determined. If drug discontinuation occurred, the date of discontinuation was estimated through chart review.

**Figure 1:**
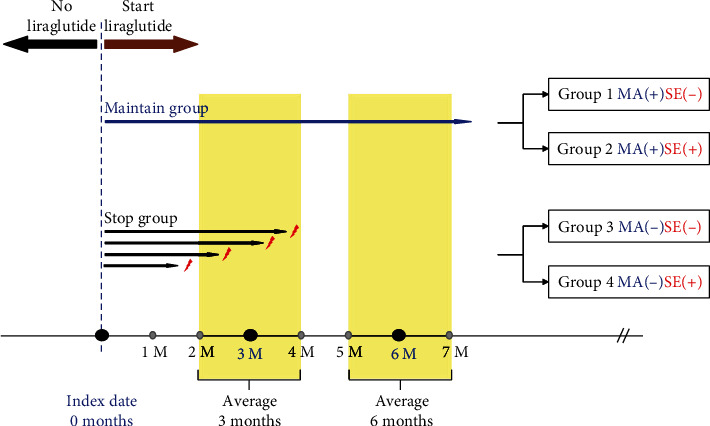
Study design.

After liraglutide was prescribed, side effects and discontinuation of the drug were checked for an average of 6 months (up to 7 months) through a direct chart review made by an endocrinologist (Figure 1). Within the group that maintained liraglutide without discontinuation, the group without side effects was designated as the MAIN_SE(-) group, and the group with side effects was designated as the MAIN_SE(+) group. We defined the time criterion for drug discontinuation as before visit 1 (2-4 months). Therefore, as per the liraglutide discontinuation criteria, only those patients who discontinued treatment within 4 months were included. Among the groups in which liraglutide was stopped, the group without side effects was designated as the STOP_SE(-) group, and the group with side effects was designated as the STOP_SE(+) group.

### 2.2. Protection of Privacy

An endocrinologist with more than 10 years of experience directly reviewed the charts and collected all related data and information for all patients. The patients' charts were reviewed directly by a lead researcher. During statistical analysis, the patients' personal identification numbers were deleted, and a random number was assigned. All personally identifiable data were anonymized and stored in various combinations. Anonymization and security measures were implemented in this study. As this was a retrospective cohort study, there was no possibility of physical or mental harm to the participants; therefore, patient consent was not required. This study was approved by the Clinical Research Ethics Committee of the Catholic Medical Center (approval number: XC20RIDI0060).

### 2.3. Statistical Analysis

Baseline variables are expressed as means and standard deviations or numbers, including percentages, whether they were continuous or categorical variables. The association between weight variations during the 3 months after prescription and the degree of discontinuation was assessed using an independent *t*-test considering the Bonferroni correction. The final regression models between weight variation and baseline variables were executed using a linear regression model adjusted for age, sex, and BMI. All statistical analyses were performed using the SAS software (SAS Institute Inc., Cary, NC, USA), and statistical significance was set at *p* < 0.05.

## 3. Results

From January 1, 2014, to December 31, 2019, 157 patients were prescribed with liraglutide at Seoul St. Mary's Hospital and Eunpyeong St. Mary's Hospital (Table 1). The mean age of the patients was 44 ± 12 years, and the proportion of female participants was 74.5% (117/157 patients), which was higher than that of male participants. The average body weight was 83.5 ± 18.0 kg, and the average BMI was 30.5 ± 4.8 kg/m^2^; there was no significant difference between the groups. For 6 months, 40.8% patients (64/157) maintained liraglutide, while 59.2% (93/157) stopped taking it. The group that stopped liraglutide stopped it at 3.3 ± 1.2 months. The mean dose of liraglutide was 2.3 ± 0.8 mg, and there was no significant difference between the groups. In the group that maintained liraglutide, 64.1% patients (41/64) had no side effects, and 35.9% (23/64) had side effects. In the group that stopped liraglutide, 67.7% patients (63/93) had no side effects and 32.3% (30/93) had side effects. History of hypertension, prediabetes, and DM did not significantly differ between the groups. In addition, the history of GI problems, such as gastroesophageal reflux disease or ulcers, showed no difference between the groups (*p* = 0.060). There was no significant difference between the groups regarding bipolar disease, sleep disorders, anxiety disorders, depression, or psychiatric treatment (*p* = 0.979). Other laboratory findings did not show any significant differences between the groups.

### 3.1. Weight Change according to the Side Effects and Discontinuation of Liraglutide

**Figure 2:**
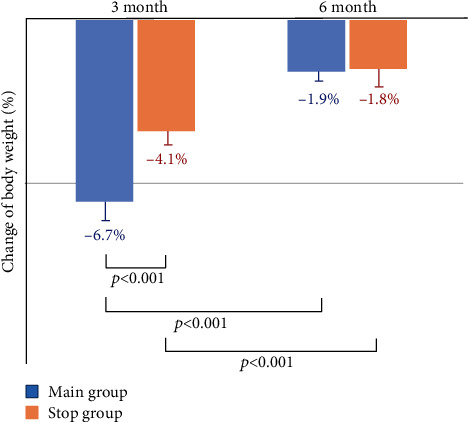
Weight change according to the discontinuation of liraglutide.

After 3 months, there was a significant weight reduction of 6.7 ± 0.5% (−5.4 ± 0.4 kg, *p* < 0.001) compared to the baseline in the MAIN group; there was a significant weight reduction of 4.1 ± 0.4% (−3.3 ± 0.3 kg, *p* < 0.001) compared to the baseline in the STOP group (Figure 2). However, the MAIN group showed greater weight reduction after 3 months than the STOP group (−6.7 ± 0.5% vs. −4.1 ± 0.4%, *p* < 0.001). After 6 months, there was a significant weight reduction of 1.9 ± 0.3% (−1.5 ± 0.3 kg, *p* < 0.001) compared to the baseline in the MAIN group and a significant weight reduction of 1.8 ± 0.5% (−1.4 ± 0.4 kg, *p* < 0.05) compared to the baseline in the STOP group. However, there was no significant difference in weight reduction between the MAIN and STOP groups after 6 months (−1.9 ± 0.3% vs. −1.8 ± 0.5%, *p* = 0.667).

**Figure 3:**
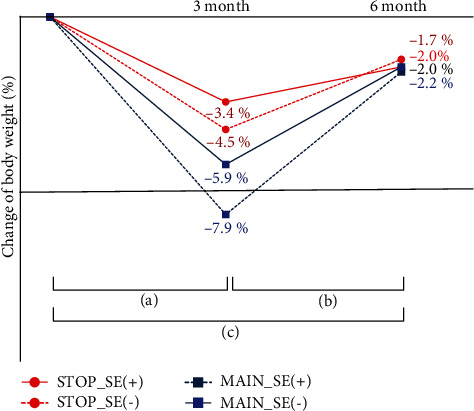
Weight change according to the side effects and discontinuation of liraglutide. (a) *p* < 0.001 at all group; (b) *p* < 0.001 at MAIN_SE(-) and *p* < 0.01 at STOP_SE(+), STOP_SE(-), and MAIN_SE(+) groups; and (c) *p* < 0.001 at all group. At 3 months, *p* < 0.01 between MAIN_SE(-) and STOP_SE(+) and *p* < 0.001 between MAIN_SE(+) and STOP_SE(-) and between MAIN_SE(+) and STOP_SE(+). At 6 months, all groups did not show statistical significance for each other.

In the detailed analysis of the presence or absence of side effects, the average weight reduction after 3 months compared to the baseline was −5.9 ± 0.6% (from 86.3 ± 19.6 kg to 81.4 ± 19.6 kg, *p* < 0.001) in the MAIN_SE(-) group, and it was −7.9 ± 0.9% (from 82.5 ± 12.5 kg to 76.1 ± 12.4 kg, *p* < 0.001) in the MAIN_SE(+) group. However, there was no significant difference between the two groups (*p* = 0.062). After an average of 6 months, the weight reduction was −2.0 ± 0.5% (from 86.3 ± 19.6 kg to 84.8 ± 19.9 kg, *p* < 0.001) in the MAIN_SE(-) group and −2.2 ± 0.7% (from 82.5 ± 12.5 kg to 80.8 ± 12.3 kg, *p* < 0.005) in the MAIN_SE(+) group; there was also no significant difference between the two groups (*p* = 0.787). After an average of 3 months, in the STOP_SE(-) group, the weight reduction compared to the baseline was −4.5 ± 0.5% (from 81.7 ± 18.3 kg to 78.2 ± 18.3 kg, *p* < 0.001), and in the STOP_SE(+) group, it was −3.4 ± 0.6% (from 84.03 ± 18.9 kg to 81.3 ± 19.1 kg, *p* < 0.001). There was no significant difference between the two groups (*p* = 0.204). After 6 months, in the STOP_SE(-) group, the weight reduction compared to the baseline was −1.7 ± 0.7% (from 81.7 ± 18.3 kg to 80.4 ± 18.4 kg, *p* = 0.008), and in the STOP_SE(+) group, it was −2.0 ± 0.6% (from 84.03 ± 18.9 kg to 82.0±19.2 kg, *p* = 0.007). There was no significant difference between the two groups (*p* = 0.694) (Figure 3). Even though there were no significant differences between the MAIN_SE(-) and MAIN_SE(+) groups and between the STOP_SE(-) and STOP_SE(+) groups, the SE(+) groups, regardless of drug maintenance, showed more weight reduction.

The greatest weight reduction in all groups was observed at 3 months, regardless of drug maintenance or side effects. However, a weight regain pattern was observed afterward. Regarding the weight difference between 3 and 6 months, the weight regain was 3.4 ± 0.3 kg (*p* < 0.001) in the MAIN_SE(-) group, 4.6 ± 0.6 kg (*p* < 0.001) in the MAIN_SE(+) group, 2.2 ± 0.5 kg (*p* < 0.001) in the STOP_SE(-) group, and 1.2 ± 0.3 kg (*p* = 0.001) in the STOP_SE(+) group. All groups had a significant weight reduction compared to the baseline at 6 months; however, their weight increased compared to that observed at 3 months with significant results (results: weight difference between 3 and 6 months in all groups).

### 3.2. Regression Model for Weight Change

Based on 3 months of discontinuation of liraglutide in the logistic regression model, factors affecting weight loss were drug discontinuation, sex, and the presence or absence of dyslipidemia (Table 2). Even after adjusting for age, sex, and BMI, which are well-known confounding variables, it was found that when liraglutide was stopped, body weight was 2.7282 times higher than when it was maintained (*p* < 0.001). Moreover, the weight loss was 1.7238 times higher in the case of dyslipidemia than in the case of no dyslipidemia (*p* < 0.01) (Supplementary Table 1).

## 4. Discussion

In our study, the presence or absence of side effects did not result in a significant difference in weight loss. The side effects did not affect the weight change. In addition, regardless of drug maintenance or side effects, the greatest weight loss was observed in all groups at 3 months, and the weight recovered gradually after that in real practice. Interestingly, in the real world, some patients tolerate and maintain liraglutide even if there are side effects. Meanwhile, there are cases in which it is stopped for various reasons, such as compliance, even if there are no side effects.

Although liraglutide has a mechanism for delayed gastric emptying, there is a weak relationship between GI symptoms and gastric emptying, considering that the symptoms occur in the fasting state [11]. It has been reported that up to 40% of patients experience nausea, and these side effects are usually mild or moderate and transient [7, 9]. Various side effects, like upper GI symptoms, such as nausea and vomiting, can lead to drug discontinuation, and randomized control studies have shown that these effects are up to 6–16% [7, 12, 13]. The result of drug discontinuation is expected to affect weight change, regardless of side effects. Since previous studies compared the side effects of liraglutide with those of placebo [14, 15], no studies have directly compared the weight loss effect of the drug discontinuation group. However, our study analyzed drug discontinuation and side effects of liraglutide individually. In our study, approximately 30% of patients complained of side effects, regardless of whether the drug was maintained or stopped. This is consistent to some extent with the fact that up to 40% of the patients complained of nausea and vomiting.

Although the presence or absence of side effects did not significantly affect the weight loss in this study, the SE(+) group showed greater weight reduction. Drug treatment is important for weight loss, as are diet and exercise [3]. As side effects from the GI tract can affect diet, the weight loss effect is expected to be greater. Some studies have shown that weight reduction is greater when people experience side effects. In one study, the weight loss was significantly greater by 2.9 kg than that in the group without side effects [7]. In our study, the SE(+)group showed more weight loss, but the effect was less than that in the previous study, and the difference due to side effects was smaller in the STOP group. These results may be related to the drug compliance.

According to the results of this study, weight reduction was greatest at around 3 months; however, there was weight regain thereafter. Notably, there was no difference in body weight between the STOP and MAIN groups at 6 months. In our study, the MAIN group lost an average of 5.4 kg (average 6.7%) at 3 months, and after recovering the body weight, it decreased by an average of 1.6 kg (average 2.0%) compared to the baseline at 6 months. There was a study in which 5.0 kg was reduced in 6 months and maintained for 5 years [16], and there was a weight loss of at least 5% after 1 year of use [1, 4, 6, 13, 17]. Although this study did not consider the 1-year period, the weight loss effect was similar at the beginning and then decreased compared to previous studies. Liraglutide is known to have a longer weight loss effect than other obesity drugs [5, 16]. However, some studies have shown weight recovery after seeing the maximum effect of the drug. Some studies also show that weight increases by 0.43 kg every year; however, it is reported that the weight loss effect is significant even when the weight is recovered [1, 6, 13, 17]. Our study also showed weight regain, and although the weight loss effect was significant, it was not as great as that of previous studies. This indicates that even after the prescription of liraglutide, the medical staff should monitor weight change while checking the patient's continued compliance.

The reason why our study had a lower weight loss effect at around 6 months compared to other studies is presumed to be a feature of real-world evidence using real-world data [18, 19]. Unlike randomized control trials, the reason why weight management is not carried out properly in real clinical fields can be presumed to be related to the compliance of the drug in the actual field, such as the inconvenience of injections, economic problems, or the presence or absence of side effects [20–22]. In addition, there are various confounding factors outside the hospital, such as lack of treatment time and adequate explanation and awareness of drugs [23]. Some studies had a pessimistic view that weight loss advice will not change the patient's behavior, lack of discipline, and motivation for the patient's own weight loss [24]. The drug discontinuation rate due to GI side effects was 6–16% in randomized control trials [7, 12, 13]. In our study, 93 patients stopped liraglutide within 6 months, accounting for more than half of the patients, and adherence was poorer. It seems that the physician and the patient were responsible for the inability to sustain weight loss in this study, regardless of compliance with liraglutide. This suggests that low adherence to liraglutide was not considered, that the prescription of liraglutide itself is not considered a serious situation, or that there is a lack of continuous education for patients after 3 months [25]. Even in this study, chart reviews by experts were performed, but in many cases, various reasons for weight loss, side effects, or drug discontinuation were not mentioned in the electronic medical records (EMR).

In our study, the factors affecting weight loss were the presence or absence of drug discontinuation and sex. In a previous study, the effect of the difference in plasma exposure of liraglutide by pharmacokinetics on the weight loss effect was investigated. Weight and sex have been identified as factors affecting the differences in plasma exposure [26]. The same BMI but lower body weight or women of any body weight showed higher plasma exposure, possibly affecting drug efficacy. However, the reason for low liraglutide clearance in women and the effects of differences in body composition and other factors have not yet been elucidated, and further research is needed in this field. In addition, socially, women will have a higher aesthetic interest than men [27], and it can be estimated that drug adherence will be high regardless of the side effects of liraglutide.

Our study had several limitations owing to its retrospective nature [28, 29]. First, the sample size was small. However, although our study population was small, it was conducted as a multicenter study so that we could control for the distribution and bias of the data. Additionally, since it was prescribed in a tertiary university hospital, it would have been relatively closer to the correct indication of liraglutide prescription than in local clinics. Furthermore, as in another retrospective study on the actual clinical effects of liraglutide in Korea with approximately 169 participants [30], there was no statistical limitation in achieving the purpose of the study. Second, confounding factors affecting body weight reduction were excluded [29]. Diet and exercise, as well as the use of other drugs, are important factors that affect the reduction in body weight; however, these factors were not considered. Additionally, it is difficult to assess drug compliance in retrospective cohort studies. However, all charts were reviewed to confirm patient compliance for an average of 6 months (up to 7 months) after liraglutide prescription. The medical chart detailed the medical staff's judgments and records of patient compliance. In addition, during this direct chart review process, the patient's visit date, prescribed dose, and number of prescriptions of liraglutide were checked to determine whether the drug was discontinued or not. This process is thought to be sufficient data to some extent to indirectly estimate patient compliance. To secure accurate data, which is a disadvantage of the retrospective study, direct chart reviews of all data were made by experts, and efforts were made to secure various variables that could affect weight loss and discontinuation to minimize bias.

The major strength of our study is that it evaluated the weight loss effect of liraglutide in an actual clinic, not in a randomized control trial, according to the presence or absence of side effects and discontinuation. All side effects of liraglutide, including those of the GI and nervous systems, were directly checked and included. The effects of these side effects on compliance, such as drug discontinuation at the treatment site, were investigated. Furthermore, this study investigated the effect of liraglutide on weight loss due to drug compliance, which is influenced by side effects.

Liraglutide is a representative drug that is maintained continuously despite its side effects because it is often used for cosmetic purposes [31]. Nevertheless, the effect of weight loss in this study did not last long. Physicians should take this cause seriously and identify its cause. The side effects of the drug are thought to cause more weight loss; however, in the real world, compliance may decrease the effect. In addition, because the weight loss effect of the drug was small in actual clinical practice and the weight was recovered even with the use of liraglutide, regardless of the presence or absence of side effects, to increase compliance, the medical staff should make an effort to manage the patient. When prescribing liraglutide, the medical staff need to continuously check whether the patient is a good fit to increase compliance. In addition, since there were many discontinuations within 3 months, it will be necessary to consider this period to ensure that the treatment can be continued. In particular, attention should be paid to many patients who discontinue liraglutide despite the absence of side effects. Although not included in this study, it is imperative to determine the reasons for discontinuing the drug (injections, pain, and discomfort). Finally, a large-scale prospective study including more diverse data on factors needed to build real-world evidence on the effects of side effects and the continued use of liraglutide is needed.

## Figures and Tables

**Table 1:** Baseline characteristics of patients (*n* = 157).

	Total	MAIN_SE(-) group	MAIN_SE(+) group	STOP_SE(-) group	STOP_SE(+) group	*p* value
Number (%)	157	41 (26.1)	23 (14.7)	63 (40.1)	30 (19.1)	
Age (years)	44 ± 12	46 ± 11	41 ± 14	43 ± 13	44 ± 12	0.486
Female sex, *n* (%)	117 (74.5)	27 (65.9)	19 (82.6)	48 (76.2)	23 (76.7)	0.461
Weight (kg)	83.5 ± 18.0	86.3 ± 20.0	82.5 ± 12.5	81.7 ± 18.3	84.0 ± 18.9	0.640
BMI (kg/m^2^)	30.5 ± 4.8	30.8 ± 4.7	30.5 ± 3.7	30.3 ± 5.4	30.3 ± 4.5	0.974
Use of liraglutide						
Period of use (month)	3.26 ± 1.24	—	—	3.25 ± 1.06	3.27 ± 1.57	0.968
Dosage (mg)	2.3 ± 0.8	2.3 ± 0.8	2.6 ± 0.6	2.3 ± 0.8	2.2 ± 0.8	0.165
Present illness						
Hypertension, *n* (%)	48 (30.6)	15 (36.6)	5 (21.7)	16 (25.4)	12 (40.0)	0.309
Prediabetes, *n* (%)	10 (6.4)	2 (4.9)	1 (4.4)	6 (9.5)	1 (3.3)	0.716
DM, *n* (%)	57 (36.3)	15 (36.6)	10 (43.5)	20 (31.8)	12 (40.0)	0.740
Dyslipidemia, *n* (%)	65 (41.4)	15 (36.6)	14 (60.9)	22 (34.9)	14 (46.7)	0.144
GI trouble history, *n* (%)	4 (2.6)	0 (0.0)	0 (0.0)	1 (1.6)	3 (10.0)	0.060
Fatty liver, *n* (%)	11 (7.0)	3 (7.3)	3 (13.0)	3 (4.8)	2 (6.7)	0.629
Thyroid disease, *n* (%)	1 (0.6)	0 (0.0)	0 (0.0)	1 (1.6)	0 (0.0)	>0.999
Psychiatric history, *n* (%)	15 (9.6)	4 (9.8)	2 (8.7)	7 (11.1)	2 (6.7)	0.979
Allergy history, *n* (%)	1 (0.6)	0 (0.0)	0 (0.0)	0 (0.0)	1 (3.3)	0.338
Laboratory finding						
Glucose (mg/dL)	113 ± 26	115 ± 27	113 ± 23	112 ± 26	110 ± 30	0.938
HbA1c (%)	6.6 ± 1.1	6.7 ± 1.0	6.8 ± 1.7	6.4 ± 1.0	6.6 ± 1.1	0.823
BUN (mg/dL)	13.4 ± 3.9	14.3 ± 4.5	12.0 ± 3.5	12.8 ± 3.7	14.1 ± 3.3	0.284
Creatinine (mg/dL)	0.8 ± 0.2	0.8 ± 0.2	0.7 ± 0.1	0.8 ± 0.2	0.7 ± 0.1	0.096
eGFR (mL/min/1.73 m^2^)	96.8 ± 21.4	91.8 ± 19.6	105.8 ± 28.1	94.4 ± 19.3	98.8 ± 21.5	0.263
AST (U/L)	35 ± 40	42 ± 60	24 ± 10	34 ± 27	39 ± 45	0.621
ALT (U/L)	45 ± 52	51 ± 66	31 ± 19	44 ± 42	53 ± 68	0.612
ALP (U/L)	56 ± 16	54 ± 13	59 ± 18	59 ± 19	55 ± 15	0.744
*γ*GTP (U/L)	44 ± 44	36 ± 22	39 ± 13	50 ± 61	44 ± 32	0.722
CPK (U/L)	102 ± 74	111 ± 60	61 ± 21	119 ± 101	80 ± 32	0.249
Total cholesterol (mg/dL)	189 ± 43	175 ± 43	192 ± 45	193 ± 43	200 ± 40	0.307
Triglycerides (mg/dL)	161 ± 95	153 ± 82	145 ± 67	154 ± 67	198 ± 162	0.402
HDL-cholesterol (mg/dL)	49 ± 13	48 ± 11	52 ± 12	48 ± 12	50 ± 17	0.779
LDL-cholesterol (mg/dL)	115 ± 40	106 ± 40	116 ± 41	120 ± 40	117 ± 41	0.669

Categorical variables are reported as frequencies (%), and continuous variables are reported as mean ± SD. AST: aspartate aminotransferase; ALT: alanine aminotransferase; ALP: alkaline phosphatase; BMI: body mass index; BUN: blood urea nitrogen; CPK: creatinine phosphokinase; DM: diabetes mellitus; eGFR: estimated glomerular filtration rate; GI: gastrointestinal; *γ*-GTP: *γ*-glutamyl transpeptidase; HbA1c: glycated hemoglobin; HDL: high-density lipoprotein; LDL: low-density lipoprotein.

**Table 2:** Regression model for weight at 3 months.

	Unadjusted		Adjusted	
	Beta estimates	*p* value	Beta estimates	*p* value
Stop liraglutide	2.5270	<0.001	2.7286	<0.001
Age	-0.0022	0.9360	-0.0071	0.7820
Male sex	-1.9235	0.0100	-2.1891	0.0020
BMI (>25 kg/m^2^)	-0.1450	0.9030	-0.8688	0.4370
Dosage (mg)	-0.7259	0.0940		
DM	0.9987	0.1430		
Hypertension	1.2880	0.0700		
Dyslipidemia	1.4426	0.0300	1.7238	0.0070

BMI: body mass index; DM: diabetes mellitus.

## Data Availability

The data that support the findings of this study are available from the corresponding author upon request.

## Supplementary Materials

Supplementary Materials Supplementary Table 1: correlation coefficient between outcome variables and baseline characteristics.
