# Supplementary material for: A Study on Weight Loss Cause as per the Side Effect of Liraglutide
Source: Cardiovasc Ther. 2022 Dec 2;2022:5201684. doi: 10.1155/2022/5201684 (PMC9733986; doi:10.1155/2022/5201684)
Supplement: Supplementary Materials — Supplementary Table 1: correlation coefficient between outcome variables and baseline characteristics. [file 5201684.f1.docx]

**Supplementary Table 1.** Correlation coefficient between outcome variables and baseline characteristics

|  | 3month (%) | Age | Sex  (male) | BMI  (>25kg/m^2^) |
| --- | --- | --- | --- | --- |
| Weight reduction at 3 month (%) |  | -0.0064 | -0.2047 | -0.0098 |
| Age | -0.0064 |  | 0.0934 | 0.0502 |
| Male sex | -0.2047 | 0.0934 |  | -0.1757 |
| BMI (>25kg/m^2^) | -0.0098 | 0.0502 | -0.1757 |  |

BMI, body mass index
